# Synthesis, Antibacterial and Insecticidal Activities of Novel Capsaicin Derivatives Containing a Sulfonic Acid Esters Moiety

**DOI:** 10.3389/fchem.2022.929050

**Published:** 2022-06-14

**Authors:** Dandan Xie, Zaiping Yang, Xin Hu, Yin Wen

**Affiliations:** ^1^ State Key Laboratory Breeding Base of Green Pesticide and Agricultural Bioengineering, Key Laboratory of Green Pesticide and Agricultural Bioengineering, Ministry of Education, Guizhou University, Guiyang, China; ^2^ School of Biology and Engineering, Guizhou Medical University, Guiyang, China; ^3^ School of Biological Sciences, Guizhou Education University, Guiyang, China

**Keywords:** sulfonic acid esters, synthesis, antibacterial activities, insecticidal activity, capsaicin derivatives

## Abstract

In order to develop an efficient and broad-spectrum bactericide, a series of novel capsaicin derivatives containing a sulfonic acid esters moiety was synthesized. The structure of these compounds were confirmed by nuclear magnetic resonance spectroscopy (NMR) and high-resolution mass spectrum (HRMS). The results of the bioactivities revealed that some target compounds exhibited remarkable antibacterial activity. Compound **3b** exhibited the highest activities against *Pseudomonas syringae* pv. *actinidiae* (Psa), *Xanthomonas oryzae* pv. *oryzae* (Xoo), and *Xanthomonas axonopodis* pv. *citri* (Xac), and the values were 86, 54, and 92% at 50 μg/ml, respectively, which were higher than were for thiodiazole copper (87, 34, and 77%) and bismerthiazol (87, 37 and 75%). Although some compounds also showed certain activity against *Spodoptera frugiperda*, it was weaker than the positive controls monosultap and mulfoxaflor. Thus, the bioassay results recommend that these newly designed and synthesized scaffolds should be used as a bactericide lead compound rather than an insecticide lead compound.

## 1 Introduction

Almost every crop is affected by bacterial diseases, resulting in significant quality and yield losses. Although there are some commercially available agricultural chemicals for bacterial disease control, frequent and long-term use of these result in problems in resistance in bacteria populations, environmental contamination, and human health. Therefore, there is still a need to develop novel, effective, and environmentally friendly bactericides (Chen et al., 2021; Li et al., 2021). The plants have established an excellent chemical defense system to selectively defend against pathogens through producing some chemicals with antimicrobial properties throughout their evolution. These secondary metabolites generally have antimicrobial activity against pathogens but are safe for the environment, animals, and humans. Thus, it could be imagined that these chemicals can be further developed into bactericides, as excellent lead structures (Morant et al., 2008a; Morant et al., 2008b; Xia et al., 2014).

**FIGURE 1:**
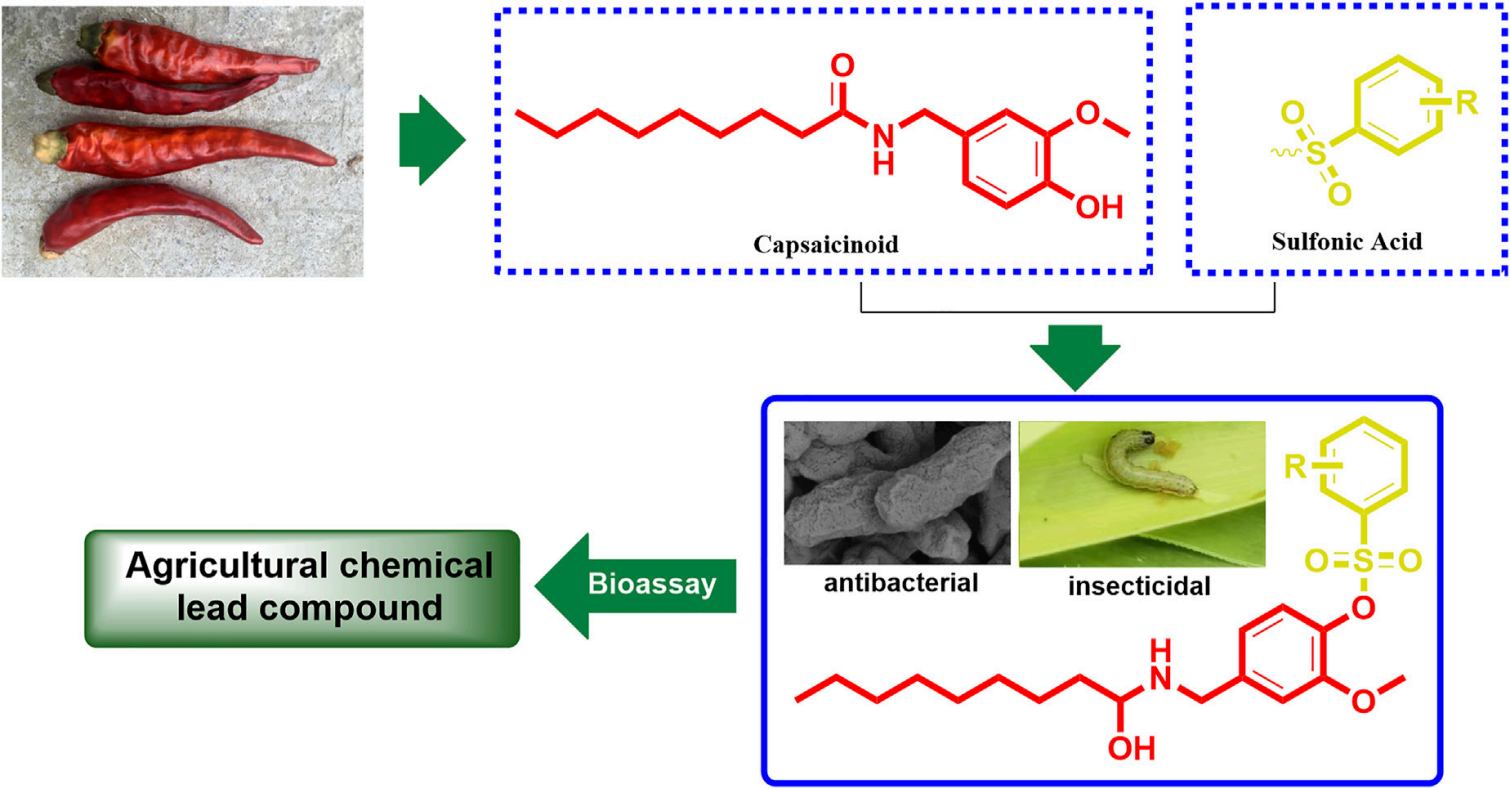
The overall design idea of research.

Capsaicinoid is originally a kind of active ingredient extracted from the ripe fruit of the nightshade plant capsicum, with more than 19 compounds having similar structures such as capsaicin, hydrocapsaicin, mocapsaicin, and nordihydrocapsaicin (Mazourek et al., 2009; Huang et al., 2013). Due to these structures being highly similar, they has almost identical biological activities, such as insecticidal, bactericidal, analgesic, anticancer, antiviral, and other such activities (Lee et al., 2007; Snitker et al., 2009; Díaz-Laviada, 2010; Liao et al., 2011). In particular, the bactericidal and insecticidal activities of capsaicinoid have been so impressive that capsaicinoid has even been developed into commercial bactericides and insecticides (Isaacs et al., 2004; Inoue et al., 2007; Claros Cuadrado et al., 2019). As a member of the capsaicinoid family, nonivamide is widely studied as a substitute of capsaicinoid in organic chemistry, analytical chemistry, and the biochemistry field, as it is easily synthesized than other capsaicinoid members (Anderson et al., 2014; Palo-Nieto et al., 2016). As the main functional group of covalent inhibitors drug, sulfonic acid groups play an important role in pharmaceutical chemistry. This is due to the sulfonic acid group easily forming covalent bonds with lysine, histidine, serine, and tyrosine of protein, which makes the parent compound better in acting on the protein target (Hatcher et al., 2018; Baggio et al., 2019; Bum-Erdene et al., 2020; Teng et al., 2020). In addition, sulfonic acid groups can also improve the physical and chemical properties of drug molecules (Guo et al., 2019; Guo et al., 2020). Inspired by the results of these studies, the present work aims to incorporate a sulfonic acid moiety into the nonivamide backbone to synthesize a series of novel derivatives and further evaluate their bactericidal and insecticidal activities (Figure 1), hoping to obtain capsaicinoid derivatives with higher activities than the existing commercial agricultural chemicals.

## 2 Experimental

### 2.1 Chemistry

All starting materials and reagents were commercially available and used without further purification, except as indicated. The ^1^H-NMR and ^13^C-NMR spectra were recorded on a Bruker DPX 400 MHz (Bruker BioSpin GmbH, Rheinstetten, Germany) NMR spectrometer with CDCl_3_ as the solvent. The following abbreviations were used to explain the multiplicities: s, singlet; d, doublet; t, triplet; m, multiplet, and br, broadened. The melting points were determined on a WRX-4 microscope melting point apparatus (YiCe Apparatus & Equipment Co., Ltd., Shanghai, China). High-resolution mass spectrometry (HRMS) was conducted using a Thermo Scientific Q Exactive (Thermo Fisher Scientific, Massachusetts, United States).

#### 2.1.1 General Procedures for Preparing Compounds

**SCHEME 1:**
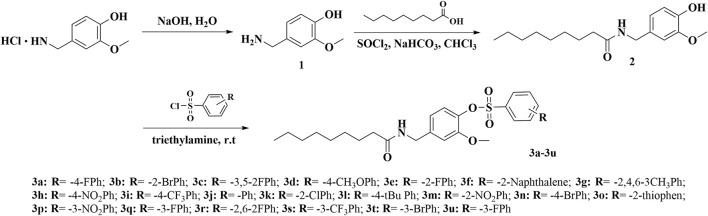
Synthetic route of the target compounds **3a**–**3u**.

The synthetic route for the title compounds **3a**–**3u** is depicted in Scheme 1. Intermediates **1–2** were synthesized according to a previous reported method (Anderson et al., 2014). Vanillylammonium chloride was dissolved in deionized water, and 10 wt% NaOH aqueous solution was added to the reaction system, slowly reaching pH = 12. Then, the white precipitate was filtered out, and sodium hydroxide solution added to the filtrate until reaching pH = 5. The intermediates **1** was precipitated from the system as a white solid. 0.12 mol of intermediates **1** and **3**–equivalent thionyl chloride was mixed and refluxed for 4 h. The excess thionyl chloride was removed by rotary evaporator, and the residue was dissolved by CH_2_Cl_2_. 0.1 mol vanillylamine was dissolved in 30 ml CH_2_Cl_2_, then a solution of acyl chloride/CH_2_Cl_2_ mixture was added dropwise to the reaction mixture. After stirring at 40°C for 6 h, the reaction was stopped, and intermediates **2** was obtained through chromatography. Target compounds **3a**–**3u** were synthesized by condensation of different sulfonyl chloride, which contains different substituent groups and intermediates **2** at room temperature conditions. Two equivalents of triethylamine was added to the system as a catalyst to neutralize the HCl generated by the reaction such that the reaction can proceed smoothly. After about approximately 4 h, the solvent was removed, and the residue was purified by flash chromatography on silica gel with petroleum *n*-hexane/ethyl acetate (volume ratio 5:1) to obtain the pure product.

##### 2.1.1.1 2-Methoxy-4-(Nonanamidomethyl)Phenyl 4-Fluorobenzenesulfonate (**3a**)

White powder, yield 81.0%. m.p. 36–37°C. ^1^H-NMR (400 MHz, CDCl_3_) *δ* 7.89–7.81 (m, 2H), 7.15 (d, *J* = 8.6 Hz, 2H), 7.06 (d, *J* = 8.0 Hz, 1H), 6.79–6.71 (m, 2H), 6.07 (t, *J* = 6.0 Hz, 1H), 4.34 (d, *J* = 6.0 Hz, 2H), 3.50 (s, 3H), 2.18 (t, *J* = 7.6 Hz, 2H), 1.61 (t, *J* = 7.3 Hz, 2H), 1.30–1.20 (m, 10H), and 0.85 (t, *J* = 6.7 Hz, 3H). ^13^C-NMR (100 MHz, CDCl_3_) *δ* 173.3, 164.6, 151.6, 139.3, 137.2, 132.1, 131.5, 131.4, 124.1, 119.6, 116.2, 116.0, 112.1, 55.5, 43.0, 36.7, 31.8, 29.3, 29.2, 25.8, 22.6, and 14.1. HRMS (ESI): calculated for C_23_H_30_FNO_5_S [M+Na]^+^: 474.1720, found: 474.1723.

##### 2.1.1.2 2-Methoxy-4-(Nonanamidomethyl)Phenyl 2-Bromobenzenesulfonate **(3b)**

White powder, yield 83.0%. m.p. 65–66.5°C. ^1^H-NMR (400 MHz, CDCl_3_) *δ* 7.98–7.77 (m, 2H), 7.54–7.34 (m, 1H), 7.19 (t, *J* = 8.6 Hz, 1H), 7.05 (dd, *J* = 36.6, 8.1 Hz, 1H), 6.86–6.65 (m, 2H), 5.96 (q, *J* = 6.3 Hz, 1H), 4.37 (t, *J* = 6.3 Hz, 2H), 3.54 (s, 3H), 2.20 (m, 2H), 1.72–1.56 (m, 2H), 1.45–1.16 (m, 10H), and 0.87 (t, *J* = 6.7 Hz, 3H). ^13^C-NMR (100 MHz, CDCl_3_) *δ* 173.2, 151.6, 135.5, 134.6, 132.1, 131.5, 131.4, 124.1, 119.6, 116.2, 116.0, 112.1, 55.6, 55.5, 43.0, 36.7, 31.8, 29.3, 29.3, 29.2, 25.8, 22.6, and 14.1. HRMS (ESI): calculated for C_23_H_30_BrNO_5_S [M+Na]^+^: 534.0920, found: 534.0921.

##### 2.1.1.3 2-Methoxy-4-(Nonanamidomethyl)Phenyl 3,5-Difluorobenzenesulfonate **(3c)**

White powder, yield 85.0%. m.p. 85.7–86.5°C. ^1^H-NMR (400 MHz, CDCl_3_) *δ* 7.62–7.35 (m, 2H), 7.16–7.05 (m, 2H), 6.91–6.67 (m, 2H), 5.85 (s, 1H), 4.40 (d, *J* = 6.0 Hz, 2H), 3.61 (s, 3H), 2.22 (t, *J* = 7.6 Hz, 2H), 1.66 (dd, *J* = 16.1, 8.8 Hz, 2H), 1.38–1.14 (m, 10H), and 0.87 (t, *J* = 6.8 Hz, 3H). ^13^C-NMR (100 MHz, CDCl_3_) *δ* 173.2, 161.2, 151.5, 139.6, 137.2, 124.0, 119.8, 112.3, 112.3, 109.6, 55.6, 43.1, 36.8, 31.8, 29.3, 29.2, 25.8, 22.6, and 14.1. HRMS (ESI): calculated for C_23_H_29_F_2_NO_5_S [M+Na]^+^: 496.1627, found: 196.1633.

##### 2.1.1.4 2-Methoxy-4-(Nonanamidomethyl)Phenyl 4-Methoxybenzenesulfonate **(3d)**

White powder, yield 81.0%. m.p. 78.9–80.2°C. ^1^H-NMR (400 MHz, CDCl_3_) *δ* 7.78 (d, *J* = 8.9 Hz, 2H), 7.06 (d, *J* = 8.7 Hz, 1H), 6.96 (d, *J* = 9.0 Hz, 2H), 6.76 (dd, *J* = 4.3, 2.4 Hz, 2H), 5.89 (s, 1H), 4.37 (d, *J* = 5.9 Hz, 2H), 3.88 (s, 3H), 3.56 (s, 3H), 2.21 (t, *J* = 7.6 Hz, 2H), 1.64 (p, *J* = 7.5 Hz, 2H), 1.38–1.18 (m, 10H), and 0.87 (t, *J* = 6.8 Hz, 3H). ^13^C-NMR (100 MHz, CDCl_3_) *δ* 173.2, 164.0, 151.9, 138.8, 137.6, 130.8, 127.5, 124.0, 119.6, 114.0, 112.1, 55.7, 55.6, 43.1, 36.8, 31.8, 29.3, 29.2, 25.8, 22.6, and 14.1. HRMS (ESI): calculated for C_24_H_33_NO_6_S [M+Na]^+^: 486.1921, found: 486.1927.

##### 2.1.1.5 2-Methoxy-4-(Nonanamidomethyl)Phenyl 2-Fluorobenzenesulfonate **(3e)**

White powder, yield 84.0%. m.p. 54.3–56.6°C. ^1^H-NMR (400 MHz, CDCl_3_) *δ* 7.84–7.74 (m, 1H), 7.70–7.62 (m, 1H), 7.28 (t, *J* = 1.3 Hz, 1H), 7.24 (td, *J* = 7.6, 1.0 Hz, 1H), 7.11 (d, *J* = 8.0 Hz, 1H), 6.78 (d, *J* = 7.6 Hz, 2H), 5.93 (s, 1H), 4.37 (d, *J* = 5.9 Hz, 2H), 3.53 (s, 3H), 2.20 (t, *J* = 7.6 Hz, 2H), 1.64 (t, *J* = 7.4 Hz, 2H), 1.27 (d, *J* = 11.3 Hz, 10H), and 0.87 (t, *J* = 6.6 Hz, 3H). ^13^C-NMR (100 MHz, CDCl_3_) *δ* 173.1, 151.7, 139.1, 137.5, 136.4, 136.3, 131.2, 124.27, 124.0, 124.0, 119.7, 117.2, 117.0, 112.1, 55.6, 43.1, 36.8, 31.8, 29.3, 29.1, 25.8, 22.6, and 14.1. HRMS (ESI): calculated for C_23_H_30_FNO_5_S [M+Na]^+^: 474.1720, found: 474.1723.

##### 2.1.1.6 2-Methoxy-4-(Nonanamidomethyl)Phenyl Naphthalene-1-Sulfonate **(3f)**

White powder, yield 82.0%. m.p. 52.5–54.0°C. ^1^H-NMR (400 MHz, CDCl_3_) *δ* 8.40 (d, *J* = 1.9 Hz, 1H), 8.02–7.84 (m, 4H), 7.75–7.61 (m, 2H), 7.09 (d, *J* = 8.2 Hz, 1H), 6.76 (dd, *J* = 8.2, 2.0 Hz, 1H), 6.72 (d, *J* = 2.0 Hz, 1H), 5.84 (s, 1H), 4.36 (d, *J* = 5.9 Hz, 2H), 3.39 (s, 3H), 2.25–2.14 (m, 2H), 1.62 (q, *J* = 7.1 Hz, 2H), 1.32–1.20 (m, 10H), and 0.86 (t, *J* = 6.8 Hz, 3H). ^13^C-NMR (100 MHz, CDCl_3_) *δ* 173.1, 151.8, 139.0, 137.6, 135.4, 133.2, 131.7, 130.3, 129.5, 129.4, 129.0, 127.9, 127.7, 124.1, 123.3, 119.6, 112.2, 55.5, 43.1, 36.8, 31.8, 29.3, 29.3, 29.1, 25.8, 22.6, and 14.1. HRMS (ESI): calculated for C_27_H_33_NO_5_S [M+Na]^+^: 506.2079, found: 506.1972.

##### 2.1.1.7 2-Methoxy-4-(Nonanamidomethyl)Phenyl 2,4,6-Trimethylbenzenesulfonate **(3g)**

White powder, yield 85.0%. m.p. 62.5–64.0°C. ^1^H-NMR (400 MHz, CDCl_3_) *δ* 7.02–6.93 (m, 3H), 6.77 (d, *J* = 2.0 Hz, 1H), 6.73 (dd, *J* = 8.2, 2.0 Hz, 1H), 5.97 (t, *J* = 6.0 Hz, 1H), 4.36 (d, *J* = 5.8 Hz, 2H), 3.55 (s, 3H), 2.56 (s, 6H), 2.32 (s, 3H), 2.24–2.07 (m, 2H), 1.62 (q, *J* = 7.3 Hz, 2H), 1.46–1.21 (m, 10H), and 0.87 (t, *J* = 6.7 Hz, 3H). ^13^C-NMR (100 MHz, CDCl_3_) *δ* 173.2, 152.1, 143.5, 140.6, 138.6, 137.5, 131.6, 131.4, 123.8, 119.5, 112.2, 55.5, 43.1, 36.7, 31.8, 29.3, 29.2, 25.8, 22.8, 22.6, 21.1, and 14.1. HRMS (ESI): calculated for C_26_H_37_NO_5_S [M+Na]^+^: 498.2285, found: 498.2282.

##### 2.1.1.8 2-Methoxy-4-(Nonanamidomethyl)phenyl 4-Nitrobenzenesulfonate **(3h)**

White powder, yield 80.0%. m.p. 65.0–67.0°C. ^1^H-NMR (400 MHz, CDCl_3_) *δ* 8.23 (dd, *J* = 109.8, 8.8 Hz, 4H), 7.15 (d, *J* = 8.2 Hz, 1H), 6.82 (dd, *J* = 8.3, 2.0 Hz, 1H), 6.79 (d, *J* = 2.0 Hz, 1H), 5.80 (s, 1H), 4.40 (d, *J* = 6.0 Hz, 2H), 3.53 (s, 3H), 2.26–2.16 (m, 2H), 1.65 (d, *J* = 3.2 Hz, 2H), 1.35–1.20 (m, 10H), and 0.87 (t, *J* = 6.8 Hz, 3H). ^13^C-NMR (100 MHz, CDCl_3_) *δ* 173.1, 151.4, 150.8, 142.0, 139.7, 137.1, 129.9, 124.1, 123.9, 119.8, 112.2, 55.5, 43.1, 36.8, 31.8, 29.3, 29.3, 29.2, 25.7, 22.6, and 14.1. HRMS (ESI): calculated for C_23_H_30_N_2_O_7_S [M+Na]^+^: 501.1666, found: 501.1669.

##### 2.1.1.9 2-Methoxy-4-(Nonanamidomethyl)phenyl 4-(Trifluoromethyl)Benzenesulfonate **(3i)**

White powder, yield 83.0%. m.p. 87.9–89.1°C. ^1^H-NMR (400 MHz, CDCl_3_) *δ* 7.90 (dd, *J* = 87.7, 8.3 Hz, 4H), 7.14 (d, *J* = 8.3 Hz, 1H), 6.81 (dd, *J* = 8.2, 2.0 Hz, 1H), 6.76 (d, *J* = 2.0 Hz, 1H), 5.82 (s, 1H), 4.39 (d, *J* = 6.0 Hz, 2H), 3.47 (s, 3H), 2.34–2.06 (m, 2H), 1.73–1.56 (m, 2H), 1.38–1.21 (m, 10H), and 0.87 (t, *J* = 6.8 Hz, 3H). ^13^C-NMR (100 MHz, CDCl_3_) *δ* 173.1, 151.5, 139.4, 137.2, 129.1, 125.9, 125.9, 125.8, 125.8, 124.2, 119.8, 112.1, 55.4, 43.1, 36.8, 31.8, 29.3, 29.3, 29.1, 25.8, 22.6, and 14.1. HRMS (ESI): calculated for C_24_H_30_F_3_NO_5_S [M+Na]^+^: 524.1689, found: 524.1731.

##### 2.1.1.10 2-Methoxy-4-(Nonanamidomethyl)Phenyl Benzenesulfonate **(3j)**

White powder, yield 82.0%. m.p. 71.2–72.7°C. ^1^H-NMR (400 MHz, CDCl_3_) *δ* 7.85 (dd, *J* = 8.4, 1.3 Hz, 2H), 7.71–7.60 (m, 1H), 7.51 (t, *J* = 7.9 Hz, 2H), 7.06 (d, *J* = 7.9 Hz, 1H), 6.87–6.67 (m, 2H), 6.05 (s, 1H), 4.36 (d, *J* = 5.9 Hz, 2H), 3.49 (s, 3H), 2.23–2.17 (m, 2H), 1.72–1.55 (m, 2H), 1.36–1.22 (m, 10H), and 0.87 (t, *J* = 6.8 Hz, 3H). ^13^C-NMR (100 MHz, CDCl_3_) *δ* 173.2, 151.8, 139.1, 137.4, 136.2, 134.0, 128.8, 128.5, 124.0, 119.6, 112.1, 55.5, 43.0, 36.7, 31.8, 29.3, 29.2, 25.8, 22.6, and 14.1. HRMS (ESI): calculated for C_23_H_31_NO_5_S [M–H]^+^: 433.1923, found: 432.1850.

##### 2.1.1.11 2-Methoxy-4-(Nonanamidomethyl)Phenyl 2-Chlorobenzenesulfonate **(3k)**

White powder, yield 85.3%. m.p. 78.2–79.8°C. ^1^H-NMR (400 MHz, CDCl_3_) *δ* 7.97–7.78 (m, 2H), 7.50–7.33 (m, 1H), 7.19 (t, *J* = 8.6 Hz, 1H), 7.10 (d, *J* = 8.0 Hz, 1H), 7.01 (d, *J* = 8.1 Hz, 2H), 6.86–6.70 (m, 2H), 5.97 (s, 1H), 4.37 (t, *J* = 6.3 Hz, 2H), 3.54 (s, 3H), 2.20 (td, *J* = 7.6, 5.0 Hz, 2H), 1.75–1.55 (m, 2H), 1.35–1.18 (m, 10H), and 0.87 (t, *J* = 6.7 Hz, 3H). ^13^C-NMR (100 MHz, CDCl_3_) *δ* 173.2, 151.8, 139.2, 137.6, 134.7, 131.9, 126.6, 124.1, 119.6, 112.2, 55.6, 43.0, 36.7, 31.8, 29.3, 29.1, 25.8, 22.6, and 14.1. HRMS (ESI): calculated for C_23_H_30_ClNO_5_S [M+Na]^+^: 490.1425, found: 490.1429.

##### 2.1.1.12 2-Methoxy-4-(Nonanamidomethyl)Phenyl 4-(Tert-Butyl)Benzenesulfonate **(3l)**

White powder, yield 83.0%. m.p. 81.2–83.0°C. ^1^H-NMR (400 MHz, CDCl_3_) *δ* 7.65 (dd, *J* = 105.8, 8.6 Hz, 4H), 7.11 (d, *J* = 8.1 Hz, 1H), 6.83–6.70 (m, 2H), 5.83 (s, 1H), 4.39 (d, *J* = 5.9 Hz, 2H), 3.49 (s, 3H), 2.21 (t, *J* = 7.6 Hz, 2H), 1.68–1.57 (m, 2H), 1.35 (s, 9H), 1.33–1.16 (m, 10H), and 0.87 (t, *J* = 6.8 Hz, 3H). ^13^C-NMR (100 MHz, CDCl_3_) *δ* 173.1, 158.0, 151.9, 138.8, 137.6, 133.2, 128.4, 125.8, 124.2, 119.6, 112.1, 55.5, 43.2, 36.8, 35.3, 31.8, 31.0, 29.3, 29.3, 29.2, 25.8, 22.6, and 14.1. HRMS (ESI): calculated for C_23_H_30_ClNO_5_S [M+Na]^+^: 512.2549, found: 512.2444.

##### 2.1.1.13 2-Methoxy-4-(Nonanamidomethyl)Phenyl 2-Nitrobenzenesulfonate **(3m)**

White powder, yield 83.2%. m.p. 88.0–89.2°C. ^1^H-NMR (400 MHz, CDCl_3_) *δ* 8.03 (dd, *J* = 8.0, 1.4 Hz, 1H), 7.90–7.80 (m, 2H), 7.72 (ddd, *J* = 8.8, 7.3, 1.7 Hz, 1H), 7.09 (d, *J* = 8.0 Hz, 1H), 6.88–6.66 (m, 2H), 5.96 (s, 1H), 4.39 (d, *J* = 6.0 Hz, 2H), 3.54 (s, 3H), 2.26–2.18 (m, 2H), 1.63 (q, *J* = 7.1 Hz, 2H), 1.35–1.21 (m, 10H), and 0.91–0.84 (m, 3H). ^13^C-NMR (100 MHz, CDCl_3_) *δ* 173.2, 151.6, 139.6, 137.4, 134.9, 132.0, 131.6, 130.2, 124.7, 124.2, 119.8, 112.4, 55.6, 43.0, 36.7, 31.8, 29.3, 29.2, 25.8, 22.6, 14.2, and 14.1. HRMS (ESI): calculated for C_23_H_30_N_2_O_7_S [M+Na]^+^: 501.1666, found: 501.1668.

##### 2.1.1.14 2-Methoxy-4-(Nonanamidomethyl)Phenyl 4-Bromobenzenesulfonate **(3n)**

White powder, yield 80.1%. m.p. 79.1–80.8°C. ^1^H-NMR (400 MHz, CDCl_3_) *δ* 7.77–7.62 (m, 4H), 7.11 (d, *J* = 8.1 Hz, 1H), 6.85–6.70 (m, 2H), 5.80 (d, *J* = 7.3 Hz, 1H), 4.39 (d, *J* = 6.0 Hz, 2H), 3.54 (s, 3H), 2.33–2.16 (m, 2H), 1.65 (d, *J* = 2.9 Hz, 3H), 1.50–1.17 (m, 10H), and 1.00–0.75 (m, 3H). ^13^C-NMR (100 MHz, CDCl_3_) *δ* 173.1, 151.6, 139.2, 137.3, 135.2, 132.1, 130.0, 129.3, 124.1, 119.7, 112.1, 55.5, 43.1, 36.8, 31.8, 29.3, 29.2, 25.8, 22.6, and 14.1. HRMS (ESI): calculated for C_23_H_30_BrNO_5_S [M+Na]^+^: 534.0920, found: 534.0921.

##### 2.1.1.15 2-Methoxy-4-(Nonanamidomethyl)Phenyl Thiophene-2-Sulfonate **(3o)**

White powder, yield 86.0%. m.p. 83.0–85.2°C. ^1^H-NMR (400 MHz, CDCl_3_) *δ* 7.72 (dd, *J* = 5.0, 1.4 Hz, 1H), 7.61 (dd, *J* = 3.8, 1.4 Hz, 1H), 7.18–7.01 (m, 2H), 6.79 (d, *J* = 7.1 Hz, 2H), 5.91 (s, 1H), 4.39 (d, *J* = 5.9 Hz, 2H), 3.60 (s, 3H), 2.22 (t, *J* = 7.6 Hz, 2H), 1.64 (q, *J* = 7.3 Hz, 2H), 1.39–1.18 (m, 10H), and 0.87 (t, *J* = 6.6 Hz, 3H). ^13^C-NMR (100 MHz, CDCl_3_) *δ* 173.2, 152.0, 139.2, 137.5, 135.6, 135.2, 134.3, 127.3, 124.0, 119.6, 112.2, 55.7, 43.1, 36.8, 31.8, 29.3, 29.2, 25.8, 22.6, and 14.1. HRMS (ESI): calculated for C_21_H_29_BrNO_5_S_2_ [M–H]^+^: 439.1487, found: 438.1414.

##### 2.1.1.16 *2-Methoxy-4-(Nonanamidomethyl)Phenyl 3-Nitrobenzenesulfonate* **(3p)**

White powder, yield 82.0%. m.p. 103.1–104.5°C. ^1^H-NMR (400 MHz, CDCl_3_) *δ* 8.77 (t, *J* = 2.0 Hz, 1H), 8.51 (ddd, *J* = 8.3, 2.2, 1.1 Hz, 1H), 8.24 (dt, *J* = 7.9, 1.4 Hz, 1H), 7.76 (t, *J* = 8.1 Hz, 1H), 7.20 (d, *J* = 8.2 Hz, 1H), 6.89–6.73 (m, 2H), 5.78 (s, 1H), 4.40 (d, *J* = 6.0 Hz, 2H), 3.55 (s, 3H), 2.29–2.13 (m, 2H), 1.71–1.62 (m, 2H), 1.34–1.23 (m, 10H), and 0.90–0.82 (m, 3H). ^13^C-NMR (100 MHz, CDCl_3_) *δ* 173.1, 151.3, 148.0, 139.7, 138.46, 137.0, 134.0, 130.1, 128.3, 124.2, 123.9, 120.0, 112.2, 55.6, 43.1, 36.8, 29.3, 29.3, 29.2, 25.8, 22.6, and 14.1. HRMS (ESI): calculated for C_23_H_30_N_2_O_7_S [M+Na]^+^: 501.1666, found: 501.1667.

##### 2.1.1.17 *2-Methoxy-4-(Nonanamidomethyl)Phenyl 3-Fluorobenzenesulfonate* **(3q)**

White powder, yield 80.5%. m.p. 58.0–60.3°C. ^1^H-NMR (400 MHz, CDCl_3_) *δ* 7.68 (d, *J* = 7.9 Hz, 1H), 7.61 (ddd, *J* = 8.0, 2.5, 1.7 Hz, 1H), 7.51 (td, *J* = 8.1, 5.2 Hz, 1H), 7.39–7.34 (m, 1H), 7.11 (d, *J* = 8.0 Hz, 1H), 6.80 (d, *J* = 7.7 Hz, 2H), 5.80 (s, 1H), 4.39 (d, *J* = 5.9 Hz, 2H), 3.56 (s, 3H), 2.29–2.16 (m, 2H), 1.87–1.56 (m, 2H), 1.42–1.19 (m, 10H), and 0.87 (t, *J* = 6.8 Hz, 3H). ^13^C-NMR (100 MHz, CDCl_3_) *δ* 173.1, 151.7, 139.2, 137.3, 130.6, 124.1, 121.3, 119.7, 115.8, 112.1, 55.6, 43.1, 36.8, 31.8, 29.3, 29.2, 25.8, 22.7, and 14.1. HRMS (ESI): calculated for C_23_H_30_FNO_5_S [M+Na]^+^: 474.1721, found: 474.1721.

##### 2.1.1.18 *2-Methoxy-4-(Nonanamidomethyl)Phenyl 2,6-Difluorobenzenesulfonate* **(3r)**

White powder, yield 82.0%. m.p. 38.2–39.1°C. ^1^H-NMR (400 MHz, CDCl_3_) *δ* 7.61 (tt, *J* = 8.5, 5.8 Hz, 1H), 7.17 (d, *J* = 8.8 Hz, 1H), 7.05 (t, *J* = 8.4 Hz, 2H), 6.81 (d, *J* = 6.8 Hz, 2H), 5.85 (s, 1H), 4.38 (d, *J* = 5.9 Hz, 2H), 3.55 (s, 3H), 2.29–2.17 (m, 2H), 1.78–1.58 (m, 2H), 1.37–1.19 (m, 10H), and 0.92–0.80 (m, 3H). ^13^C-NMR (100 MHz, CDCl_3_) *δ* 173.2, 151.5, 139.4, 137.4, 136.0, 124.2, 119.9, 113.0, 112.7, 112.2, 55.6, 43.1, 36.8, 31.8, 29.3, 29.2, 25.8, 22.7, and 14.1. HRMS (ESI): calculated for C_23_H_29_F_2_NO_5_S [M+Na]^+^: 492.1627, found: 492.1626.

##### 2.1.1.19 *2-Methoxy-4-(Nonanamidomethyl)Phenyl 3-(Trifluoromethyl)Benzenesulfonate* **(3s)**

White powder, yield 81.0%. m.p. 84.3–85.4°C. ^1^H-NMR (400 MHz, CDCl_3_) *δ* 8.17 (s, 1H), 8.06 (dt, *J* = 8.1, 1.4 Hz, 1H), 7.91 (d, *J* = 7.8 Hz, 1H), 7.67 (t, *J* = 7.9 Hz, 1H), 7.19 (d, *J* = 8.2 Hz, 1H), 6.82 (dd, *J* = 8.2, 2.0 Hz, 1H), 6.76 (s, 1H), 5.79 (s, 1H), 4.40 (s, 2H), 3.48 (s, 3H), 2.21 (t, *J* = 7.6 Hz, 2H), 1.83–1.48 (m, 2H), 1.39–1.23 (m, 10H), and 0.87 (t, *J* = 6.7 Hz, 3H). ^13^C-NMR (100 MHz, CDCl_3_) *δ* 173.1, 151.4, 139.5, 137.2, 129.5, 124.3, 119.8, 112.0, 55.3, 43.1, 36.8, 31.8, 29.3, 29.2, 25.8, 22.6, and 14.1. HRMS (ESI): calculated for C_24_H_30_F_3_NO_5_S [M+Na]^+^: 524.1689, found: 524.1691.

##### 2.1.1.20 *2-Methoxy-4-(Nonanamidomethyl)Phenyl 3-Bromobenzenesulfonate* **(3t)**

White powder, yield 86.2%. m.p. 57.5–59.5°C. ^1^H-NMR (400 MHz, CDCl_3_) *δ* 8.05 (t, *J* = 1.9 Hz, 1H), 7.78 (dt, *J* = 8.1, 2.0 Hz, 2H), 7.38 (t, *J* = 8.0 Hz, 1H), 7.13 (d, *J* = 8.1 Hz, 1H), 6.84–6.69 (m, 2H), 5.90 (s, 1H), 4.38 (d, *J* = 5.9 Hz, 2H), 3.55 (s, 3H), 2.32–2.16 (m, 2H), 1.65 (h, *J* = 7.4, 6.6 Hz, 2H), 1.40–1.20 (m, 10H), and 0.87 (t, *J* = 6.8 Hz, 3H). ^13^C-NMR (100 MHz, CDCl_3_) *δ* 173.2, 151.5, 139.3, 137.9, 137.2, 137.0, 131.4, 130.2, 127.1, 124.2, 122.6, 119.7, 112.1, 55.5, 43.1, 36.8, 31.8, 29.3, 29.2, 25.8, 22.7, and 14.1. HRMS (ESI): calculated for C_23_H_30_BrNO_5_S [M+Na]^+^: 534.0920, found: 534.0920.

##### 2.1.1.21 *2-Methoxy-4-(Nonanamidomethyl)Phenyl 3-Chlorobenzenesulfonate* **(3u)**

White powder, yield 83.5%. m.p. 66.6–68.4°C. ^1^H-NMR (400 MHz, CDCl_3_) *δ* 7.90 (t, *J* = 1.9 Hz, 1H), 7.80–7.69 (m, 1H), 7.66–7.57 (m, 1H), 7.45 (t, *J* = 8.0 Hz, 1H), 7.13 (d, *J* = 8.1 Hz, 1H), 6.87–6.72 (m, 2H), 5.79 (s, 1H), 4.39 (d, *J* = 5.9 Hz, 2H), 3.55 (s, 3H), 2.40–2.01 (m, 2H), 1.79–1.52 (m, 2H), 1.35–1.21 (m, 10H), and 0.87 (t, *J* = 6.8 Hz, 3H). ^13^C-NMR (100 MHz, CDCl_3_) *δ* 173.1, 151.6, 139.3, 137.8, 137.3, 135.0, 134.1, 130.0, 128.6, 126.7, 124.2, 119.8, 112.1, 55.5, 43.1, 36.8, 31.8, 29.3, 29.2, 25.8, 22.7, and 14.1. HRMS (ESI): calculated for C_23_H_30_ClNO_5_S [M+H]^+^: 467.1553, found: 468.1604.

### 2.2 Antimicrobial Assay

The bacteria which was used for the bioassay was provided by the Guizhou Tea Institute. The test method which was reported by Wang et al. (2021) was adopted, and the commercial agricultural bactericide bismerthiazol and thiodiazole copper were used as the positive control. Compounds were diluted to a concentration using a small amount of dimethyl sulfoxide (DMSO) and 0.1% Tween-20 (v/v). The bacteria were first grown in nutrient broth medium (NB), and then the medium containing the bacteria was added to solvent NB containing the test compounds. The inoculated test tubes were incubated at 30 ± 1°C under continuous shaking at 180 rpm for 48 h. The culture growth was monitored spectrophotometrically by measuring the optical density at 600 nm (OD_600_) and expressed as corrected turbidity. The relative inhibition rates (%) were calculated according to the following equation, where C_tur_ is the corrected turbidity value of bacterial growth in untreated NB, and T_tur_ is the corrected turbidity value of bacterial growth in treated NB.

$$Inhibition\;\left(\%\right)\;=\;\left(\;C_{tur}\;-\;T_{tur}\;\right)\;/\;C_{tur}\;\times \;100\%.$$

### 2.3 Insecticidal Activity Assay

*Spodoptera frugiperda* used in the biological tests were collected from the fields in Luodian County, Guizhou Province, China, and bred in a greenhouse. The specific test steps are as follows: 20 s instar larvae of *Spodoptera frugiperda* were divided into 20 small cups and starved for 3–4 h. Fresh corn leaves were cut into small leaf disks of 1 cm × 1 cm with scissors, and then soaked in each test solution for 5 s and air dried naturally. Then, they were put in the cups with *Spodoptera frugiperda* and kept under conditions of 25 ± 1°C, relative humidity of 60–70%, and a light-dark cycle of L:D = 14 h:10 h. Normal fresh corn leaf disks were given as feed after 12 h and the number of dead insects recorded at 12, 24, and 36 h.

## 3 Results and Discussion

### 3.1 Chemistry

The synthesis of intermediates **1** and **2** was achieved according to the method reported in the literature (Anderson et al., 2014). Target compounds were synthesized by condensation of different sulfonyl chloride which contained different substituent groups and intermediates **2** at room temperature conditions. HCl was produced as the byproduct in this reaction, and it was necessary to add an alkali to neutralize the HCl in the system to make the reaction proceed smoothly. Initially, we used Na_2_CO_3_ as the catalyst to be added to the system, but because Na_2_CO_3_ was difficult to dissolve in organic systems, the reaction was slow and the product yield was also very low. Therefore, we chose organic base triethylamine as the catalyst for the reaction, which made the reaction faster, and the yield was generally higher than 80%. The structures of all title compounds were confirmed by ^1^H-NMR, ^13^C-NMR, and HRMS. Due to these compounds having similar backbones, their ^1^H-NMR spectra had something in common. For example, the proton signals of -OCH_3_ of all compounds were around 3.5 ppm, and the proton signals of acetyl were between 5.0 and 6.0 ppm. The proton signals of the alkyl chain in capsaicin were all below 4.0 ppm and were reflected in the NMR as four types of signals. These were the characteristics of the NMR spectra of these compounds.

### 3.2 *In vitro* Antibacterial Activity

As shown in Table 1, almost all title compounds exhibited antibacterial activities against *Pseudomonas syringae* pv. *actinidiae* (Psa), *Xanthomonas oryzae* pv. *oryzae* (Xoo), and *Xanthomonas axonopodis* pv. *citri* (Xac). Although many compounds exhibited comparable inhibition rates against Psa and Xac at a high concentration (100 μg/ml) as commercial bactericides, some compounds exhibited greater activity at a lower concentration (50 μg/ml). The inhibition rate of these compounds against Psa was in the range of 71–96% at 50 μg/ml. Among them, compounds **3c**, **3t**, and **3u** showed the highest activity with inhibition rates of 89, 96, and 93%, respectively, which were better than those of commercial bactericide bismerthiazol (87%) and thiodiazole copper (87%). The inhibition rate of these compounds against Xac was in the range of 71–92% at 50 μg/ml. Among them, compounds **3b** and **3p** showed the highest activity with inhibition rates of 92%, which were better than those of commercial bactericide bismerthiazol (77%) and thiodiazole copper (75%). The title compounds exhibited impressive inhibition activity against Xoo, especially compounds **3a**, **3b**, **3c**, **3d**, **3f**, **3g**, **3i**, and **3l**, and the bactericidal activity at the concentration of 100 and 50 μg/ml was higher than that in the commercial bactericide.

**TABLE 1:** The *in vitro* antibacterial activities of the target compounds **3a**–**3u**.

Compounds	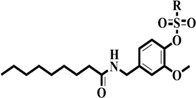	*Pseudomonas syringae* pv. *actinidiae* (Psa)		*Xanthomonas oryzae* pv. *oryzae* (Xoo)		*Xanthomonas axonopodis* pv. *citri* (Xac)	
	R	100 *μ*g/ml	50 *μ*g/ml	100 *μ*g/ml	50 *μ*g/ml	100 *μ*g/ml	50 *μ*g/ml
**3a**	-4-FPh	100 ± 3.0	83 ± 2.3	98 ± 2.4	40 ± 1.3	99 ± 1.1	81 ± 1.0
**3b**	-2-BrPh	100 ± 1.4	86 ± 3.0	94 ± 2.8	54 ± 2.6	100 ± 1.6	92 ± 3.3
**3c**	-3,5-2FPh	98 ± 0.9	89 ± 2.7	84 ± 2.4	46 ± 1.7	98 ± 1.6	77 ± 1.1
**3d**	-4-CH_3_OPh	100 ± 6.4	83 ± 3.9	90 ± 3.4	43 ± 2.7	98 ± 2.5	77 ± 2.1
**3e**	-2-FPh	100 ± 0.4	88 ± 2.9	61 ± 1.7	48 ± 2.2	99 ± 1.7	81 ± 1.3
**3f**	-2-Naphthalene	100 ± 2.2	74 ± 1.2	88 ± 3.9	52 ± 1.9	100 ± 1.6	71 ± 1.7
**3g**	-2,4,6-3CH_3_Ph	100 ± 1.7	78 ± 1.6	92 ± 1.6	49 ± 2.3	96 ± 1.5	73 ± 2.7
**3h**	-4-NO_2_Ph	100 ± 0.2	77 ± 2.2	59 ± 2.5	32 ± 2.3	100 ± 2.9	77 ± 3.4
**3i**	-4-CF_3_Ph	97 ± 0.5	79 ± 2.8	83 ± 1.4	50 ± 1.7	95 ± 2.3	80 ± 1.3
**3j**	-Ph	100 ± 3.4	84 ± 2.0	76 ± 2.4	42 ± 1.3	99 ± 1.8	81 ± 1.6
**3k**	-2-FPh	100 ± 2.2	80 ± 2.4	81 ± 3.1	42 ± 2.2	99 ± 1.5	76 ± 3.0
**3l**	-4-tBu Ph	100 ± 7.1	78 ± 2.6	84 ± 2.4	56 ± 1.6	98 ± 2.5	79 ± 2.9
**3m**	-2-NO_2_Ph	100 ± 1.3	79 ± 2.6	79 ± 1.5	39 ± 1.2	100 ± 2.2	81 ± 3.8
**3n**	-4-BrPh	100 ± 2.1	75 ± 2.2	82 ± 2.9	25 ± 1.5	99 ± 1.6	80 ± 1.9
**3o**	-2-Thiophene	100 ± 2.9	75 ± 2.3	86 ± 1.5	22 ± 1.4	105 ± 1.1	75 ± 2.1
**3p**	-3-NO_2_Ph	100 ± 4.6	85 ± 2.7	66 ± 1.7	54 ± 2.8	100 ± 6.0	92 ± 3.6
**3q**	-3-FPh	98 ± 0.5	77 ± 1.9	67 ± 0.6	45 ± 1.0	99 ± 1.2	71 ± 1.9
**3r**	-2,6-2FPh	97 ± 0.4	71 ± 1.8	78 ± 2.3	33 ± 0.4	100 ± 4.0	65 ± 2.8
**3s**	-3-CF_3_Ph	100 ± 0.8	84 ± 2.3	65 ± 1.3	37 ± 1.5	100 ± 3.4	80 ± 2.1
**3t**	-3-BrPh	100 ± 0.7	96 ± 2.5	79 ± 1.1	38 ± 1.7	100 ± 2.7	81 ± 2.1
**3u**	-3-ClPh	100 ± 2.7	93 ± 1.9	85 ± 2.3	34 ± 2.5	100 ± 3.3	88 ± 1.6
Bismerthiazol		100 ± 2.9	87 ± 2.6	75.8 ± 2.6	34 ± 1.2	100 ± 5.2	77 ± 1.1
Thiodiazole copper		100 ± 4.4	87 ± 3.4	79.4 ± 2.6	31 ± 1.8	100 ± 7.2	75 ± 2.5

### 3.3 Insecticidal Activity Assay Against *Spodoptera frugiperda*

**FIGURE 2:**
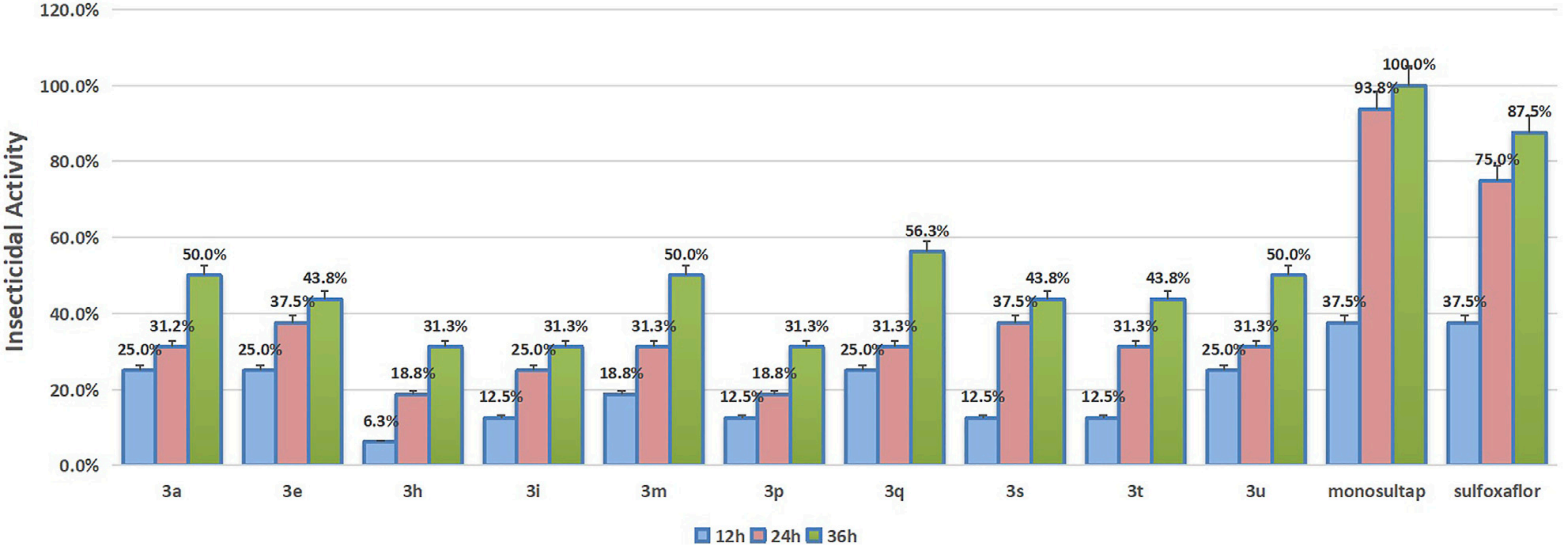
The insecticidal activity against *Spodoptera frugiperda* of target compounds.

The results of the insecticidal activity against *Spodoptera frugiperda* of some compounds which were tested are shown in Figure 2. In general, although these compounds showed certain insecticidal activity against *Spodoptera frugiperda*, their activities were lower than those of the commercial insecticide monosultap and mulfoxaflor. Compounds **3a**, **3m**, **3q**, and **3u**, which showed the highest activity among these compounds, with lethal rates of 50, 50, 56.3, and 50% at 36 h, respectively, were still far lower than the positive controls monosultap (100% at 36 h) and mulfoxaflor (87.5% at 36 h). From the structure–activity relationship analysis, the group and its substitution position on the benzene ring of the title compounds affected insecticidal activity. When the nitro group was substituted at the 2-position of the benzene ring, the compound showed enhanced insecticidal activity. When the nitro group was substituted at the 3- or 4-position, the compound showed weak insecticidal activity.

## 4 Conclusion

In summary, in order to develop efficient and broad-spectrum agricultural chemicals, we diversified the structure of capsaicin and synthesized a series of novel capsaicin derivatives containing a sulfonic acid esters moiety. The structures of the compounds were confirmed through NMR and HRMS. The bioassay results revealed that the compounds exhibited obvious activities against *Pseudomonas syringae* pv. *actinidiae* (Psa), *Xanthomonas oryzae* pv. *oryzae* (Xoo), and *Xanthomonas axonopodis* pv. *citri* (Xac). A few of the compounds even exhibited higher activities than the commercial bactericides bismerthiazol and thiodiazole copper. Surprisingly, some compounds also showed some insecticidal activity against *Spodoptera frugiperda*, but the activity was far less than that of the commercial insecticides monosultap and mulfoxaflor. Therefore, further derivatives and optimization of the structure of the title compounds to improve their insecticidal activity are underway.

## Data Availability

The original contributions presented in the study are included in the article/Supplementary Material; further inquiries can be directed to the corresponding author.
